# Elevated atmospheric CO_2_ levels affect community structure of rice root-associated bacteria

**DOI:** 10.3389/fmicb.2015.00136

**Published:** 2015-02-20

**Authors:** Takashi Okubo, Dongyan Liu, Hirohito Tsurumaru, Seishi Ikeda, Susumu Asakawa, Takeshi Tokida, Kanako Tago, Masahito Hayatsu, Naohiro Aoki, Ken Ishimaru, Kazuhiro Ujiie, Yasuhiro Usui, Hirofumi Nakamura, Hidemitsu Sakai, Kentaro Hayashi, Toshihiro Hasegawa, Kiwamu Minamisawa

**Affiliations:** ^1^Environmental Biofunction Division, National Institute for Agro-Environmental SciencesTsukuba, Japan; ^2^Department of Environmental Life Sciences, Graduate School of Life Sciences, Tohoku UniversitySendai, Japan; ^3^Division of Bioresource Functions, Graduate School of Bioagricultural Sciences, Nagoya UniversityNagoya, Japan; ^4^Large-scale Farming Research Division, Hokkaido Agricultural Research Center, National Agriculture and Food Research OrganizationHokkaido, Japan; ^5^Carbon and Nutrient Cycles Division, National Institute for Agro-Environmental SciencesTsukuba, Japan; ^6^Department of Agricultural and Environmental Biology, Graduate School of Agricultural and Life Sciences, The University of TokyoTokyo, Japan; ^7^Division of Plant Sciences, National Institute of Agrobiological SciencesTsukuba, Japan; ^8^Agro-Meteorology Division, National Institute for Agro-Environmental SciencesTsukuba, Japan; ^9^Taiyo-Keiki Co., Ltd.Tokyo, Japan

**Keywords:** rice, root, 16S rRNA gene, FACE, methane

## Abstract

A number of studies have shown that elevated atmospheric CO_2_ ([CO_2_]) affects rice yields and grain quality. However, the responses of root-associated bacteria to [CO_2_] elevation have not been characterized in a large-scale field study. We conducted a free-air CO_2_ enrichment (FACE) experiment (ambient + 200 μmol.mol^−1^) using three rice cultivars (Akita 63, Takanari, and Koshihikari) and two experimental lines of Koshihikari [chromosome segment substitution and near-isogenic lines (NILs)] to determine the effects of [CO_2_] elevation on the community structure of rice root-associated bacteria. Microbial DNA was extracted from rice roots at the panicle formation stage and analyzed by pyrosequencing the bacterial 16S rRNA gene to characterize the members of the bacterial community. Principal coordinate analysis of a weighted UniFrac distance matrix revealed that the community structure was clearly affected by elevated [CO_2_]. The predominant community members at class level were Alpha-, Beta-, and Gamma-proteobacteria in the control (ambient) and FACE plots. The relative abundance of Methylocystaceae, the major methane-oxidizing bacteria in rice roots, tended to decrease with increasing [CO_2_] levels. Quantitative PCR revealed a decreased copy number of the methane monooxygenase (*pmoA*) gene and increased methyl coenzyme M reductase (*mcrA*) in elevated [CO_2_]. These results suggest elevated [CO_2_] suppresses methane oxidation and promotes methanogenesis in rice roots; this process affects the carbon cycle in rice paddy fields.

## Introduction

Atmospheric concentration of carbon dioxide, ([CO_2_]), is expected to continue to rise during the next several decades (Fisher et al., 2007). Many studies have been conducted to understand the effects of elevated [CO_2_] on rice (e.g., yield and grain quality; Hasegawa et al., 2013; Usui et al., 2014) and on the paddy field ecosystem (e.g., methane emission; Tokida et al., 2010, 2011) using free-air [CO_2_] enrichment (FACE). Understanding the bacterial response to increased [CO_2_] is necessary to predict its effects on the rice and paddy ecosystem because rice-associated bacteria produce plant hormones, fix nitrogen, and oxidize methane (Bao et al., 2014; Ikeda et al., 2014). The relative abundances and activities of these bacteria are affected by field management (Bao et al., 2013, 2014; Ikeda et al., 2014), rice genotype (Sasaki et al., 2013; Okubo et al., 2014a), and growth stage (Okubo et al., 2014b).

It has been reported that elevated [CO_2_] levels affect the community structures and/or abundances of microorganisms in rhizosphere of grassland (Hayden et al., 2012), cropland (Schortemeyer et al., 1996), and marsh (Lee et al., 2015). Since [CO_2_] in soil is much higher than in the atmosphere, it is likely that these changes were indirectly induced by elevated [CO_2_] through increased root growth and changes of the quality and quantity of root exudates (Drigo et al., 2008). Elevated [CO_2_] significantly increases root biomass and total organic carbon in rice root exudates (Bhattacharyya et al., 2013), which may influence the activity of rhizospheric and root-associated bacteria. We previously reported that the bacterial community associated with roots and shoots of Koshihikari (a widely planted rice cultivar in Japan) might be affected by increasing [CO_2_] (Okubo et al., 2014b; Ikeda et al., in press). However, we could not draw strong conclusions at the time due to limited sample size. Meanwhile, many other studies have shown that the effects of [CO_2_] elevation on rice yield and grain quality differ between cultivars (Hasegawa et al., 2013; Myers et al., 2014; Usui et al., 2014), suggesting that the response of rice-associated bacteria also differs.

In the present study, we assessed the effects of elevated [CO_2_] on community structure in the root-associated bacteria of five rice genotypes: Akita 63 (Mae et al., 2006), Takanari (Taylaran et al., 2009), Koshihikari, a chromosome segment substitution line (CSSL) of Koshihikari that carries chromosomal segment from Kasalath for increasing the grain number (designated as CSSL-Gn1; Madoka et al., 2008), and a near-isogenic line (NIL) of Koshihikari that carries chromosomal segments from Kasalath containing the sucrose phosphate synthase gene (designated as NIL-SPS1; Hashida et al., 2013). Akita 63 and Takanari tend to produce a greater yield enhancement rate as a result of [CO_2_] elevation than does Koshihikari (Hasegawa et al., 2013). The productivity of CSSL-Gn1 and NIL-SPS1 is greater than that of Koshihikari under ambient [CO_2_] levels (Madoka et al., 2008; Hashida et al., 2013). These traits make these rice genotypes suitable for managing the growing demand for food by the world's growing population.

## Materials and methods

### Study site

The study was conducted during the 2012 growth season as part of an ongoing rice FACE study at Tsukubamirai, Ibaraki, Japan (35°58′27″N, 139°59′32″E, 10 m above sea level). The soil of the experimental site is fluvisol, which is typical in alluvial areas. Bulk density is 0.87 × 10^6^ g.m^−3^. Total C and N content is 21.4 and 1.97 mg.g^−1^, respectively. Cation exchange capacity is 202 μmolc.g^−1^ (Hasegawa et al., 2013). The experimental site was established in 2010, and the control protocols for FACE were described previously (Nakamura et al., 2012). Briefly, four rice paddy fields were used as replicates, each with two areas at ambient levels of CO_2_ (AMBI) and elevated [CO_2_] (FACE). Each treatment area was a 240-m^2^ octagon (hereafter “a ring”). The FACE rings had emission tubes on all eight sides that released pure CO_2_ from the windward sides to maintain a stable concentration at the ring's center. The CO_2_ level was set to 200 μmol.mol^−1^ above the ambient concentration (Nakamura et al., 2012). The AMBI and FACE rings were separated by at least by 70 m (center to center), which is sufficient to prevent cross-contamination by CO_2_ (Heim et al., 2009).

### Rice cultivation and fertilization

We tested five rice (*Oryza sativa* L.) genotypes: Akita 63, Takanari, Koshihikari, CSSL-Gn1, and NIL-SPS1. The CSSL-Gn1 (Madoka et al., 2008) carries a chromosomal segment of Kasalath on the Koshihikari genetic background to increase the grain number; the substituted region is located on chromosome 1 approximately between restriction fragment length polymorphism (RFLP) markers R687 and C178 (Ebitani et al., 2005). The NIL-SPS1 (Hashida et al., 2013) carries two chromosomal segments of Kasalath on chromosome 1 containing *OsSPS1* (1.1 centimorgans) and chromosome 10 (4.1 centimorgans) on the genetic background of Koshihikari. Rice was sown on April 24, 2012 in seedling trays with 448 cells (Minoru Pot 448, Minoru Industrial Co., Ltd., Okayama, Japan). Three seeds were sown in each cell. After emergence, we raised the seedlings in a puddled open field with a tunnel cloche or floating mulch for the first two weeks. On May 23 and 24, seedlings at the five-leaf stage were manually transplanted into the rings, at three seedlings per hill (“hill” is a group of seedlings transplanted to one spot). Hills and rows were 15 and 30 cm apart, respectively, with a resultant density of 22.2 hills.m^−2^. Fertilizers were applied as basal dressing. Phosphate and potassium were added on April 9 as a compound fertilizer (Sumitomo Chemical Co., Ltd., Tokyo, Japan) containing 4.36 (g P).m^−2^ and 8.30 (g K).m^−2^. Nitrogen was added on May 14 at 8 g.m^−2^ (2 and 6 g.m^−2^ as urea and coated urea, respectively; 4 g of LP-100 and 2 g of LP-140; JCAM-Agri Co., Ltd., Tokyo, Japan). The method of rice cultivation and fertilization was as described previously (Hasegawa et al., 2013). Immediately after the nitrogen application, the field was puddled for uniformity on May 17, 2012.

### Rice sampling and microbial DNA preparation

Plants were collected from three hills from each treatment plot on July 18 and 19, 2012 (56–57 days after transplanting), corresponding to the panicle formation stage. At each hill, a block of plow layer soil (30 cm length × 15 cm length × 15 cm depth) was taken with the plants and immediately transported to the laboratory. The soil was washed away with tap water and the roots were separated from the aboveground parts and stored at −80°C. The root samples were manually ground to a fine powder in liquid nitrogen using a mortar and pestle. Three ground-root samples collected from the same ring of the same genotypes and treatment (FACE or AMBI) were composited and homogenized in a blender. Microbial cells including endophytes and epiphytes were extracted by density gradient ultracentrifugation as described (Ikeda et al., 2009). Total DNA was prepared as described (Ikeda et al., 2009).

### 16S rRNA gene sequence analysis

These genes were amplified as follows: 10 ng total bacterial DNA was used as a template in a final reaction volume of 50 μL including 0.1 μM of each primer and 2 U of Ex Taq DNA polymerase (Takara Bio, Shiga, Japan) with the universal primers 27F (5′-AGAGTTTGATCMTGGCTCAG-3′) and 518R (5′-TTACCGCGGCTGCTGG-3′), containing the 454 FLX adaptors and a sample-specific multiplex identifier (Okubo et al., 2012; Ikeda et al., 2014). The cycling conditions were as follows: initial denaturation for 2 min at 94°C; 25 cycles of 30 s at 94°C, 30 s at 55°C, and 1.5 min at 72°C; and the final extension step of 8 min at 72°C. PCR products of the predicted size (~500 bp) were purified using the Wizard SV Gel and PCR Clean-Up System (Promega Japan, Tokyo, Japan). Sequencing was performed on 454 GS FLX+ (Roche Diagnostics K.K., Tokyo, Japan). The pyrosequencing reads were processed using the Quantitative Insights Into Microbial Ecology (QIIME) software package (Caporaso et al., 2010). The sequences were assigned to each sample according to the sample-specific multiplex identifier. Low-quality sequences shorter than 300 bp, with an average quality score lower than 25, with mismatching primer sequences, or with ambiguous bases (marked as “N”), were eliminated from downstream analyses. The forward and reverse primer regions were removed from the quality-filtered sequences. Potentially chimeric sequences were removed using the USEARCH6.1 software (Edgar, 2010). Potentially contaminated sequences classified as *chloroplast*, *mitochondria*, or unassigned by the RDP Classifier software (Wang et al., 2007) were removed. The remaining sequences were clustered into operational taxonomic units (OTUs) at 97% similarity using the pick_de_novo_otus command with default parameters. Principal coordinates analysis (PCoA) was performed on weighted and unweighted UniFrac distance matrixes (Lozupone and Knight, 2005) using a random sample of 2000 sequences for data normalization. For statistical testing, to determine the effects of the [CO_2_] elevation, rice genotype, and their interaction, permutational multivariate analysis of variance (PERMANOVA) was conducted on the UniFrac distance matrixes using the *adonis* function in the R software package vegan (http://vegan.r-forge.r-project.org/). The numbers of OTUs and Chao1 as well as Shannon, and Simpson's indexes were calculated with 10 replicates using a random sample of 2000 sequences for data normalization. Statistical analysis was performed on the mean values of 10 replicates to determine the effects of the [CO_2_] elevation, rice genotype, and their interaction using linear mixed model of the SPSS Statistics software, version 22 (IBM Japan, Tokyo, Japan). [CO_2_] and rice genotype were treated as fixed effects, while field and field × [CO_2_] were treated as random effects. The phylogenetic composition of the sequences was evaluated using the RDP classifier (Wang et al., 2007), with confidence levels of 80%. Statistical analysis was also performed on the relative abundance of each taxonomic group to determine the effects of the [CO_2_] elevation, rice genotype, and their interaction.

### Quantification of *pmoA* and *mcrA* genes

The copy numbers of *pmoA* and *mcrA* in the microbial DNA were determined using a Thermal Cycler Dice Real Time System (TaKaRa, Shiga, Japan) with primers A189f/mb661r (Holmes et al., 1995; Costello and Lidstrom, 1999) for the *pmoA* gene and mcrA-f/mcrA-r (Luton et al., 2002) for the *mcrA* gene. For both genes, reactions were performed in a total volume of 25 μL containing 12.5 μL SYBR Premix ExTaq, 0.1 μL each primer (50 mM), 10 ng template DNA, and 12.3 μL sterilized ultrapure water. The PCR conditions were as follows: 40 cycles of denaturation at 95°C for 30 s, annealing at 65.5°C for 30 s, and extension at 72°C for 45 s for *pmoA* and 45 cycles of denaturation at 95°C for 40 s, annealing at 55°C for 30 s, and extension at 72°C for 60 s for *mcrA*. Clones of the *pmoA* genes derived from *Methylosinus trichosporium* strain OB3b (GenBank accession number U31650) and *Methylomonas koyamae* strain Fw12E-Y (GenBank accession number AB538965) were used to generate a standard curve for the quantification of *pmoA* gene copies. For the quantification of *mcrA* gene copies, *mcrA* gene fragments derived from *Methanobrevibacter arboriphilus* strain SA (GenBank accession number AB300777), *Methanosarcina mazei* strain TMA (GenBank accession number AB300778), and *Methanoculleus chikugoensis* strain MG62 (GenBank accession number AB300779) were used to construct a standard curve. The copy numbers of *pmoA* and *mcrA* were processed by means of linear mixed model in the SPSS Statistics software, version 22. Outliers were excluded from the statistical analysis.

### The nucleotide sequence accession number

Raw sequence data were deposited in the DNA Data Bank of Japan (DDBJ) Sequence Read Archive under accession number: DRA002644.

## Results

### Richness and diversity indices of a bacterial community

**Figure 1:**
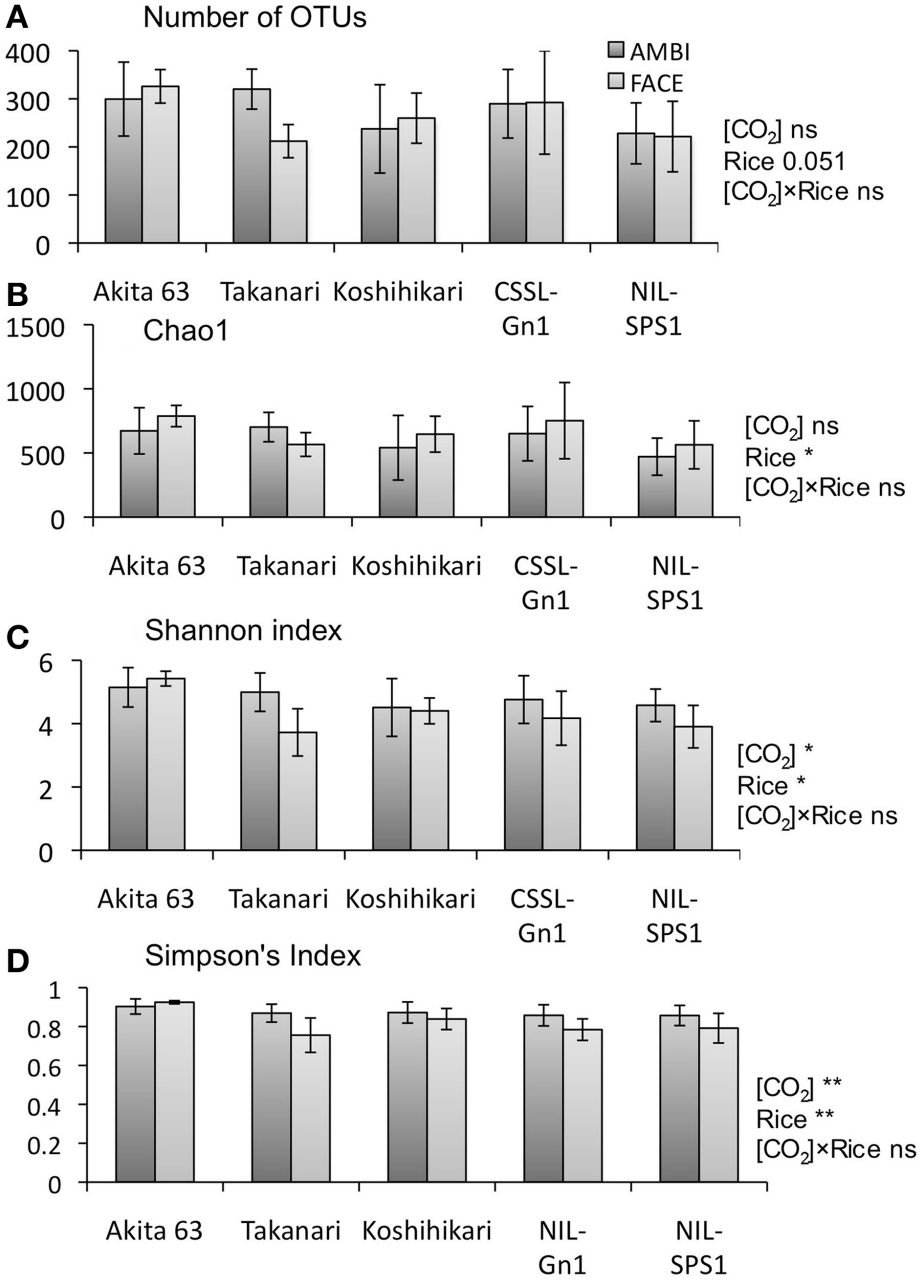
**The effects of [CO_2_] elevation and rice cultivar on the richness and diversity indices of a root-associated bacterial community (*n* = 4)**. **(A)** Number of OTUs; **(B)** Chao1; **(C)** Shannon index; **(D)** Simpson's index. Error bars represent standard deviation. Statistically significant effects are indicated: ^**^p < 0.01 and ^*^p < 0.05. The value indicates the probability between 0.05 and 0.1; ns, not significant (*p* > 0.1); OTU, operational taxonomic unit; AMBI, ambient levels of CO_2_; CSSL, chromosome segment substitution line; FACE, free-air CO_2_ enrichment; NIL, near-isogenic line.

The number of 16S rRNA gene sequences analyzed in the present study is shown in Table S1. The rarefaction curves for the number of OTUs are shown in Figure S1. Richness and diversity of a root-associated bacterial community were evaluated using the 16S rRNA gene sequences with the number of OTUs and Chao1 as well as Shannon and Simpson's (1 − Dominance) indices. The effects of the [CO_2_] elevation were not statistically significant with regard to the number of OTUs (*p* = 0.1, Figure 1A) and Chao1 (*p* = 0.1, Figure 1B), suggesting that the [CO_2_] elevation had little or no effect on the richness of root-associated bacterial communities. Shannon (Figure 1C) and Simpson's (Figure 1D) indices were significantly decreased by the [CO_2_] elevation (*p* < 0.05), indicating that the [CO_2_] elevation decreased the diversity of root-associated bacterial communities. The [CO_2_] × rice genotype interaction was not statistically significant for all indices (Figure 1, *p* = 0.1), suggesting that bacterial communities associated with rice roots showed similar responses to the [CO_2_] elevation regardless of genotype. The effect of rice genotype was almost significant for all indices (Figure 1, *p* = 0.051).

### An overview of bacterial community structures

**Figure 2:**
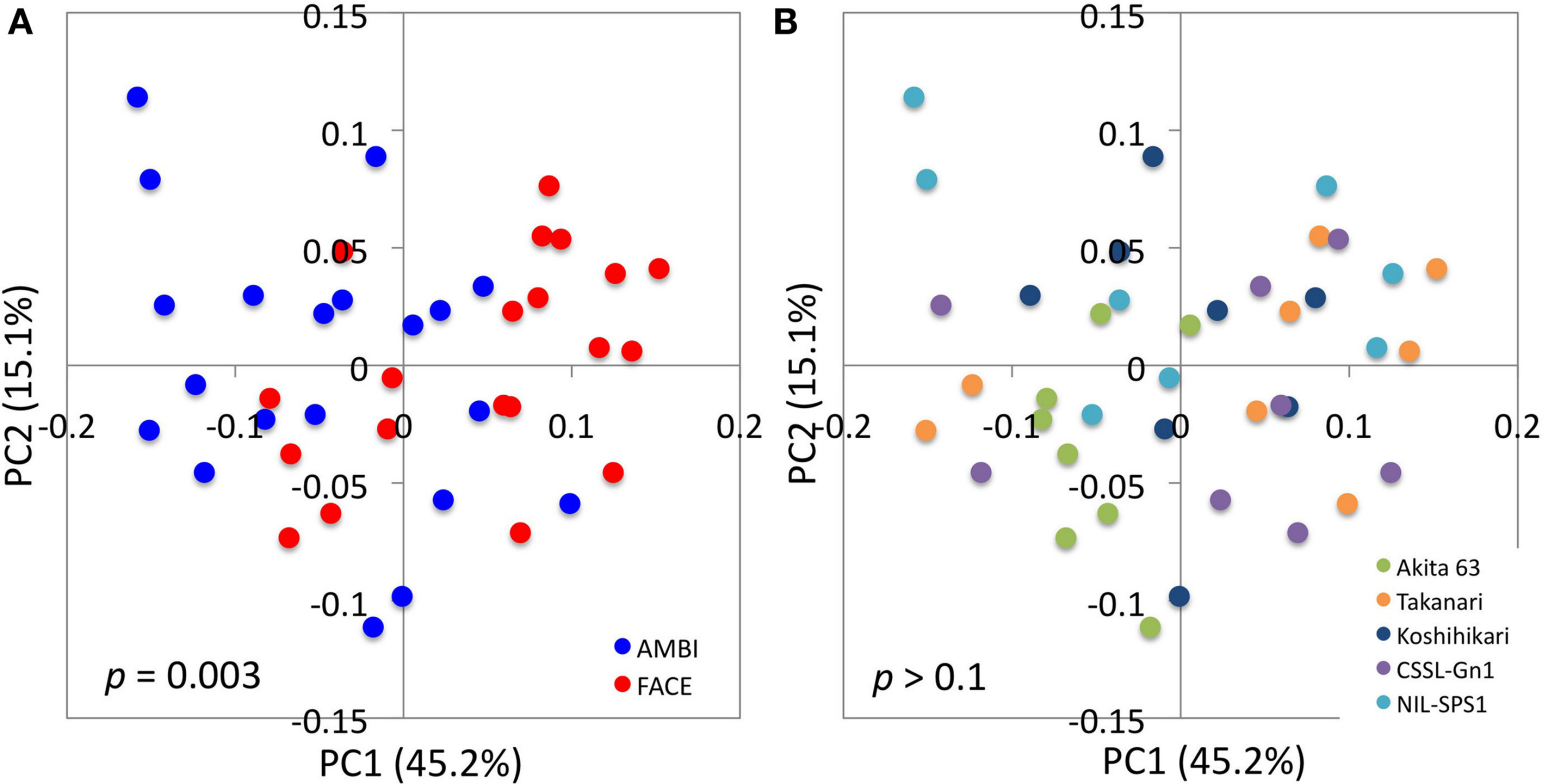
**UniFrac principal coordinates analysis plots illustrating the effects of the [CO_2_] elevation and rice cultivar on the structure of root-associated bacterial communities**. Distance matrices were defined by a weighted UniFrac distance. Data points are colored according to [CO_2_] treatment in **(A)** or by rice cultivar in **(B)**. The results of statistical tests of differences between treatments are indicated in each plot. The [CO_2_] × rice genotype interaction was not statistically significant (*p* > 0.1). PC, principal component; AMBI, ambient levels of CO_2_; CSSL, chromosome segment substitution line; FACE, free-air CO_2_ enrichment; NIL, near-isogenic line.

PCoA based on weighted and unweighted UniFrac distance matrices was performed to obtain an overview of changes in the bacterial community structure caused by [CO_2_] elevation, rice genotype, and [CO_2_] × rice genotype interaction. In the weighted UniFrac analysis, the [CO_2_] elevation separated samples along the axis of the first principal component (PC1; Figure 2A). The adonis test yielded a *p* value of 0.003, indicating that the community structures of root-associated bacteria were significantly affected by the [CO_2_] elevation. In contrast, the effects of rice genotype (Figure 2B) and [CO_2_] × rice genotype interaction (Figures 2A,B) on community structure were not statistically significant (*p* = 0.1). Statistical analysis suggested that the changes in a bacterial community caused by the [CO_2_] elevation represented a general response of rice plants, regardless of genotype. Akita 63, however, tended to show a smaller response to the [CO_2_] elevation, compared to the other rice genotypes (Figure 2B and Figure S2). In unweighted UniFrac analysis, the [CO_2_] elevation (Figure S3A), rice genotype (Figure S3B), and [CO_2_] × rice genotype interaction (Figures S3A,B) had no significant effect on community structure (*p* = 0.1). These results suggest that [CO_2_] elevation affected the relative abundance of bacterial species rather than which bacterial species were present or absent because unweighted UniFrac analysis ignores the information on abundance of OTUs and takes into account only data on the presence and absence of OTUs.

### Phylogenetic composition

**Figure 3:**
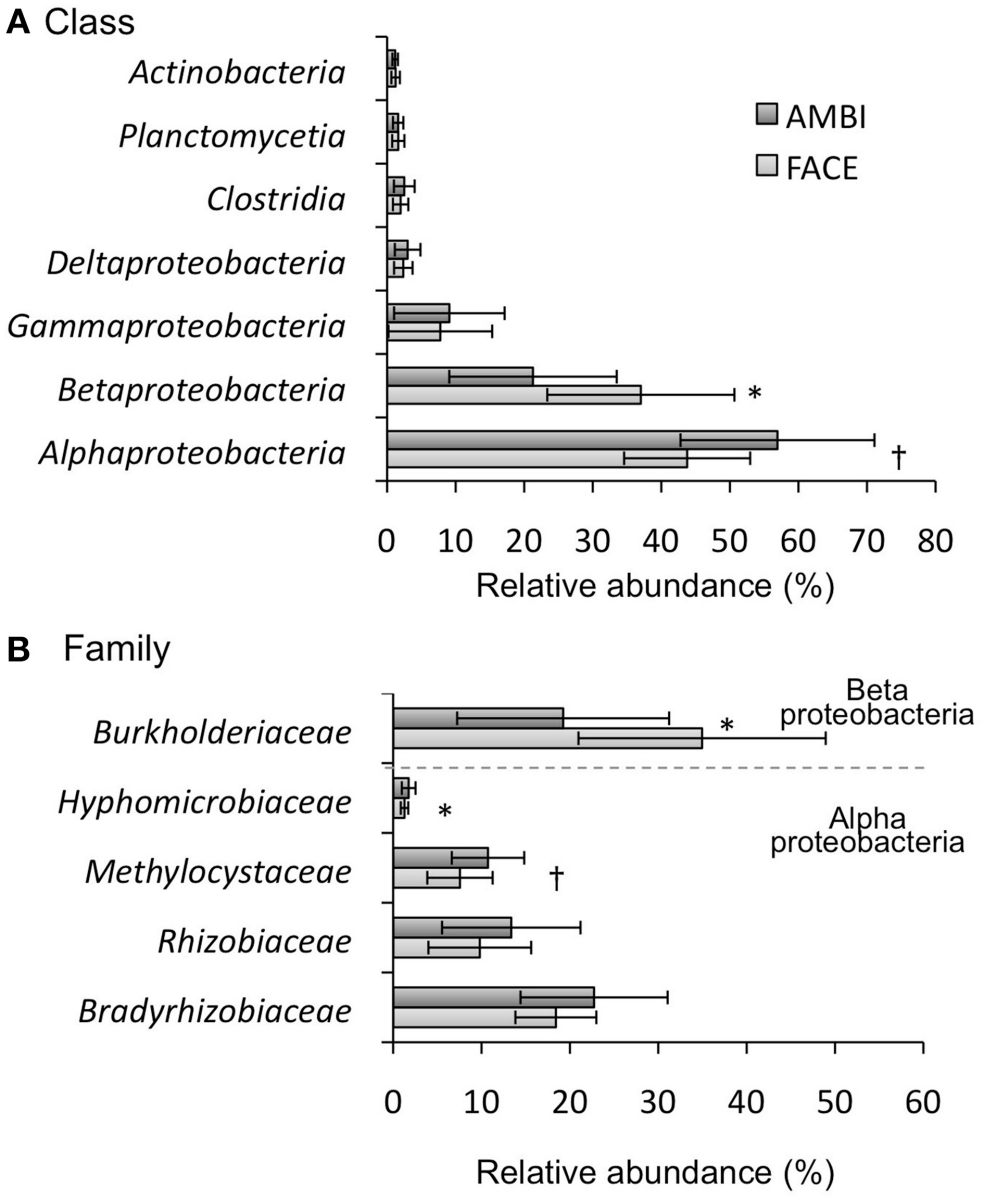
**Phylogenetic composition of root-associated bacteria at the class level in (A) and at the family level in (B) (*n* = 4)**. The relative abundance is shown in averages for five rice genotypes. Error bars represent standard deviation. Statistically significant effects of the [CO_2_] elevation: ^*^p < 0.05 and ^†^*p* < 0.1. AMBI, ambient levels of CO_2_; FACE, free-air CO_2_ enrichment.

Class-level data on rice root-associated bacterial communities are depicted in Figure 3A and Table S2. Alphaproteobacteria (38.0–70.0%) and Betaproteobacteria (16.0–48.9%) were the predominant classes followed by Gammaproteobacteria, Deltaproteobacteria, Clostridia, Planctomycetia, and Actinobacteria. The relative abundance of Alphaproteobacteria tended to be reduced by the [CO_2_] elevation (Figure 3A, Table S2; *p* = 0.085), whereas the Betaproteobacteria increased (Figure 3A, Table S2; *p* < 0.05). Other classes were not clearly affected by the [CO_2_] elevation (Figure 3A, Table S2; *p* = 0.1). The effect of [CO_2_] on the relative abundance of Alphaproteobacteria and Betaproteobacteria was not observed in Akita 63 (Table S2). At the family level, Burkholderiaceae were predominant, representing 13.7–46.5% of all sequences (Figure 3B, Table S3). The relative abundance of Burkholderiaceae was significantly increased by the [CO_2_] elevation (Figure 3B, Table S3; *p* < 0.05). In Alphaproteobacteria, the families of Bradyrhizobiaceae (15.5–30.8%), Rhizobiaceae (5.6–15.2%), and Methylocystaceae (5.4–13.0%) were predominant (Figure 3B, Table S3); their relative abundance showed a decreasing trend by the [CO_2_] elevation. Although the decreases of Bradyrhizobiaceae and Rhizobiaceae were not statistically significant (*p* = 0.1), those might be due to the limited sample size in our study, given the large standard deviation (Figure 3B). In all samples, Methylocystaceae were the dominant methane-oxidizing bacteria under both AMBI and FACE conditions, representing 5.4–13.0% of all sequences (Figure 3B, Table S3), followed by Methylophilaceae (0–0.1%) and Methylococcaceae (0–0.05%). It is worth mentioning that Methylacidiphilales, which are often found in acidic geothermal environments (Sharp et al., 2014), were also detected in root-associated bacterial communities, with a relative abundance of 0–0.05% under both AMBI and FACE conditions.

### Copy numbers of the *pmoA* and *mcrA* genes

**Figure 4:**
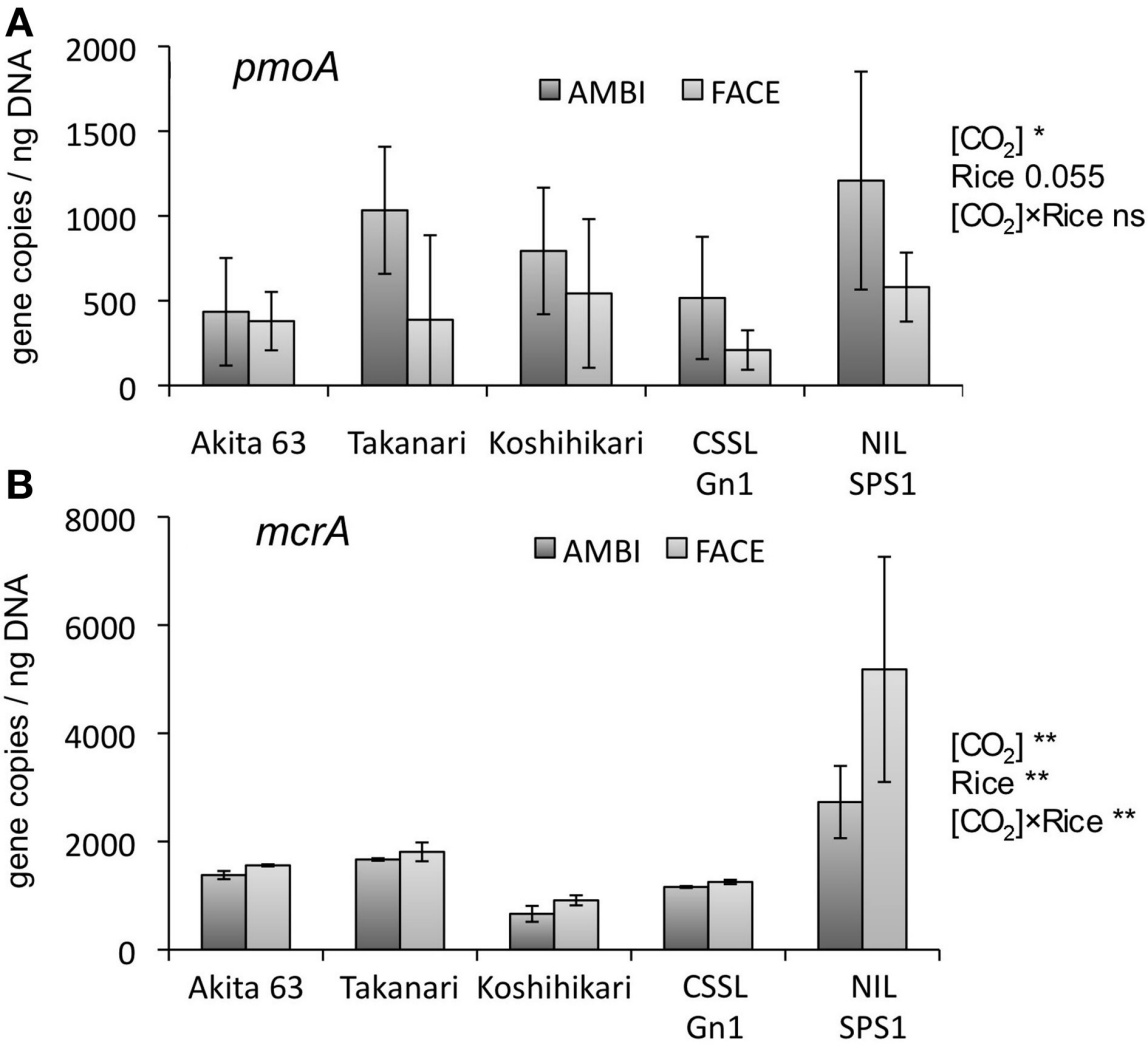
**The effects of the [CO_2_] elevation and rice cultivar on *pmoA* (A) and *mcrA* (B) gene copy number in a root-associated bacterial community (*n* = 4, except *n* = 3 for the *pmoA* copy number of Akita 63)**. Error bars show standard deviation. Statistically significant effects are indicated: ^**^p < 0.01, and ^*^p < 0.05. The value indicates the probability between 0.05 and 0.1; ns, not significant (*p* > 0.1); AMBI, ambient levels of CO_2_; CSSL, chromosome segment substitution line; FACE, free-air CO_2_ enrichment; NIL, near-isogenic line.

QPCR was performed to estimate methane monooxygenase (*pmoA*) and methyl coenzyme M reductase (*mcrA*) gene copy number in the DNA of microbial communities extracted from rice roots. The copy number of *pmoA* was significantly reduced by the [CO_2_] elevation (Figure 4A, *p* < 0.01). The effect of the [CO_2_] × rice genotype interaction was not statistically significant in relation to the *pmoA* copy number (Figure 4A, *p* = 0.1). The effect of rice genotype on *pmoA* copy number was marginally significant (*p* = 0.055). NIL-SPS1 tended to show a higher copy number than CSSL-Gn1 (Figure 4A). Compared to Koshihikari, the average *pmoA* copy number was higher in NIL-SPS1 but lower in CSSL-Gn1, suggesting that those substituted genomic regions had the opposite effect on the relative abundance of root-associated methane-oxidizing bacteria. The *mcrA* copy number was significantly increased by [CO_2_] elevation (Figure 4B, *p* < 0.01). The effect of the rice genotype on the copy number of *mcrA* was also significant (Figure 4B, *p* < 0.01). NIL-SPS1 showed a significantly higher *mcrA* copy number than the other rice genotypes (*p* < 0.001). Although the *mcrA* copy number showed a trend for an increase in all rice genotypes under the influence of [CO_2_] elevation, the rate of increase differed among the genotypes (a significant [CO_2_] × rice genotype interaction, *p* < 0.01). For example, in NIL-SPS1, this copy number was increased by 89.9%, whereas in CSSL-Gn1 and Koshihikari, the copy number increased only by 7.9 and 37.7%, respectively.

## Discussion

The structure of root-associated bacterial communities is clearly affected by an increase in [CO_2_] (Figure 2A). The results of PCoA based on weighted (Figure 2A) and unweighted (Figure S3A) UniFrac distances suggest that the [CO_2_] elevation affects the relative abundance of bacteria rather than which bacterial linages are present or absent in rice roots. Shannon (Figure 1C) and Simpson's (Figure 1D) indices show that the [CO_2_] elevation significantly reduces bacterial diversity. This decrease could be partially explained by the very high relative abundance of Burkholderiaceae (14.9–46.5%) under elevated [CO_2_] (Figure 3B and Table S3). In contrast, the [CO_2_] elevation had little or no effect on the richness of a bacterial community, according to the number of OTUs (Figure 1A) and Chao1 (Figure 1B). Thus, the results on richness and diversity indices are consistent with the UniFrac PCoA plots with regard to the responses to [CO_2_] elevation.

The effects of the [CO_2_] × rice genotype interaction on UniFrac PCoA plots (Figure 2 and Figure S3) and on the richness and diversity indices of a bacterial community (Figure 1) are not statistically significant, suggesting that bacterial communities associated with rice roots show similar responses to [CO_2_] regardless of the genotype. Nevertheless, the effects of the [CO_2_] × rice genotype interaction need to be examined in more detail because the bacterial community associated with Akita 63 tends to show a smaller response to the [CO_2_] elevation than the bacterial communities associated with the other rice genotypes (Figure 2 and Figure S2). The statistically insignificant effects of the [CO_2_] × rice genotype interaction might be due to the limited sample sizes, considering the large standard variations that we observed (Figure S2).

The copy number of *pmoA* (Figure 4A) and the relative abundance of Methylocystaceae (the dominant methanotrophs in rice roots; Figure 3B) were reduced by the [CO_2_] elevation. The decrease in the relative abundance of Methylocystaceae was observed in another FACE study (Okubo et al., 2014b). These results suggest the methane-oxidizing activity in rice roots is downregulated by [CO_2_] elevation. In contrast, the copy number for *mcrA* was significantly increased by the [CO_2_] elevation (Figure 4B), suggesting that the abundance of methanogenic Archaea associated with rice roots increases under elevated [CO_2_]; this change may lead to upregulation of methanogenesis in rice roots. Other FACE studies showed that the elevated [CO_2_] stimulates photosynthesis in plants (Sasaki et al., 2005). Additional assimilated carbon under elevated [CO_2_] increases the carbon content of root exudates (a potential carbon source for root-associated microorganisms; Bhattacharyya et al., 2013); this change may increase the abundance of root-associated methanogenic Archaea. The present study demonstrates that the copy number of *mcrA* is higher in NIL-SPS1 than in Koshihikari. One study showed that the SPS activity in NIL-SPS1 is higher than that in Koshihikari at the panicle formation stage (Hashida et al., 2013). It is likely that the SPS activity correlates with the amount of the sucrose pool available for transport (Huber, 1983). The difference in photosynthetic carbon allocation between NIL-SPS1 and Koshihikari may influence *mcrA* copy number in root-associated microbial communities.

We originally hypothesized that the copy number of *pmoA* correlates with that of *mcrA* because methane is the major substrate for methanotrophs. Nevertheless, the copy number of *pmoA* was reduced by the [CO_2_] elevation, whereas *mcrA* copy number increased (Figures 4A,B). These results are suggestive of the existence of other factors limiting the population of methanotrophs in rice roots. Other FACE studies revealed that [CO_2_] elevation decreases N and Cu concentrations in rice plants (Myers et al., 2014). These chemical elements are known to be important for methane oxidation (Semrau et al., 2010). If N and Cu are less available for rice root-associated bacteria under elevated [CO_2_], then this situation may decrease the rate of methane oxidation in rice roots, leading to a decrease in the relative abundance of methane-oxidizing bacteria.

Although our results showed that [CO_2_] elevation shifts the community structure of rice root-associated bacteria, the mechanism of that shift remains unclear. Previous studies reported that elevated [CO_2_] increases the carbon content of root exudates (Bhattacharyya et al., 2013). This increase will affect the community, activity, and food webs of rhizosphere microorganisms, which may contribute to the community shift of root-associated bacteria. Thus, ecological complexities in rhizosphere processes make it difficult to produce a straightforward explanation.

In the present study, we described a shift in the community structure of rice root-associated bacteria under the influence of [CO_2_] elevation. There are still some challenges to elucidating the secondary effects of the changes in bacterial community structure on the rice yield and paddy ecosystem. Changes in the relative abundance of methanotrophs and methanogens will affect the carbon cycle in a rice paddy. In addition, metaproteomic analysis of rice root-associated bacteria revealed that Methylocystaceae are typical nitrogen-fixing bacteria in rice roots (Bao et al., 2014). The [CO_2_] elevation may reduce the amount of N_2_ fixed by Methylocystaceae in rice roots; this change is expected to aggravate the N deficiency in rice under elevated [CO_2_], in addition to the dilution effect due to the greater production of dry matter.

As far as we know, ours is the first study to compare the structure of root-associated bacterial communities with the copy number analysis of microbial functional genes in NIL, CSSL, and their recurrent parent. Although statistically significant community differences were not observed by PCoA analysis (Figures 2A,B), the copy number of *pmoA* (Figure 4A) and *mcrA* (Figure 4B) varied, suggesting genomic substitution affects the activity of root-associated microorganisms. Over the past few decades, quantitative trait loci and chromosomal regions affecting traits have been screened for genes that enhance the physiological and morphological contributors to rice productivity (Madoka et al., 2008; Ujiie et al., 2012). NILs and CSSLs are also powerful tools for elucidating the genetic basis underlying the influence of rice plants on the activity of the associated microorganisms. Analysis of chromosomal regions affecting traits in the associated microbial communities should allow us to develop new cultivars that accommodate microorganisms that are more beneficial for the plants.

### Conflict of interest statement

The authors declare that the research was conducted in the absence of any commercial or financial relationships that could be construed as a potential conflict of interest.

## Abbreviations

AMBI: ambient levels of CO_2_
CSSL: chromosome segment substitution line
FACE: free-air CO_2_ enrichment
NIL: near-isogenic line
OUT: operational taxonomic unit
PCoA: principal coordinates analysis
[CO_2_]: atmospheric CO_2_ concentration.
