# Supplementary material for: Elevated atmospheric CO2 levels affect community structure of rice root-associated bacteria
Source: Front Microbiol. 2015 Feb 20;6:136. doi: 10.3389/fmicb.2015.00136 (PMC4335179; doi:10.3389/fmicb.2015.00136)
Supplement: Supplementary file 1 [file Image1.PDF]

Supplementary Material

Elevated atmospheric CO_2 levels affect community structure of rice root-associated bacteria

Takashi Okubo[1,2], Dongyan Liu[3], Hirohito Tsurumaru[2], Seishi Ikeda[4], Susumu Asakawa[3], Takeshi Tokida[1], Kanako Tago[1], Masahito Hayatsu[1], Naohiro Aoki[5], Ken Ishimaru[6], Kazuhiro Ujiie[6], Yasuhiro Usui[1], Hirofumi Nakamura[7], Hidemitsu Sakai[1], Kentaro Hayashi[1], Toshihiro Hasegawa[1], and Kiwamu Minamisawa[2*]

Author affiliations:

[1]National Institute for Agro-Environmental Sciences, Tsukuba, Ibaraki, Japan

[2]Graduate School of Life Sciences, Tohoku University, Sendai, Miyagi, Japan

[3]Graduate School of Bioagricultural Sciences, Nagoya University, Nagoya, Aichi, Japan

[4]Memuro Research Station, National Agricultural Research Center for Hokkaido Region, Kasaigun, Hokkaido, Japan

[5]Graduate School of Agricultural and Life Sciences, The University of Tokyo, Bunkyo-ku, Tokyo, Japan

[6]National Institute of Agrobiological Sciences, Tsukuba, Ibaraki, Japan

[7]Taiyo-Keiki Co., Ltd., Kita-ku, Tokyo, Japan

*Correspondence: Dr. Kiwamu Minamisawa, Graduate School of Life Sciences, Laboratory of Environmental Plant Microbiology, Tohoku University, Katahira 2-1-1, Aoba-ku, Sendai, Miyagi 980-8577, Japan

kiwamu@ige.tohoku.ac.jp

1. Supplementary Figures and Tables

1.1. Supplementary Figures

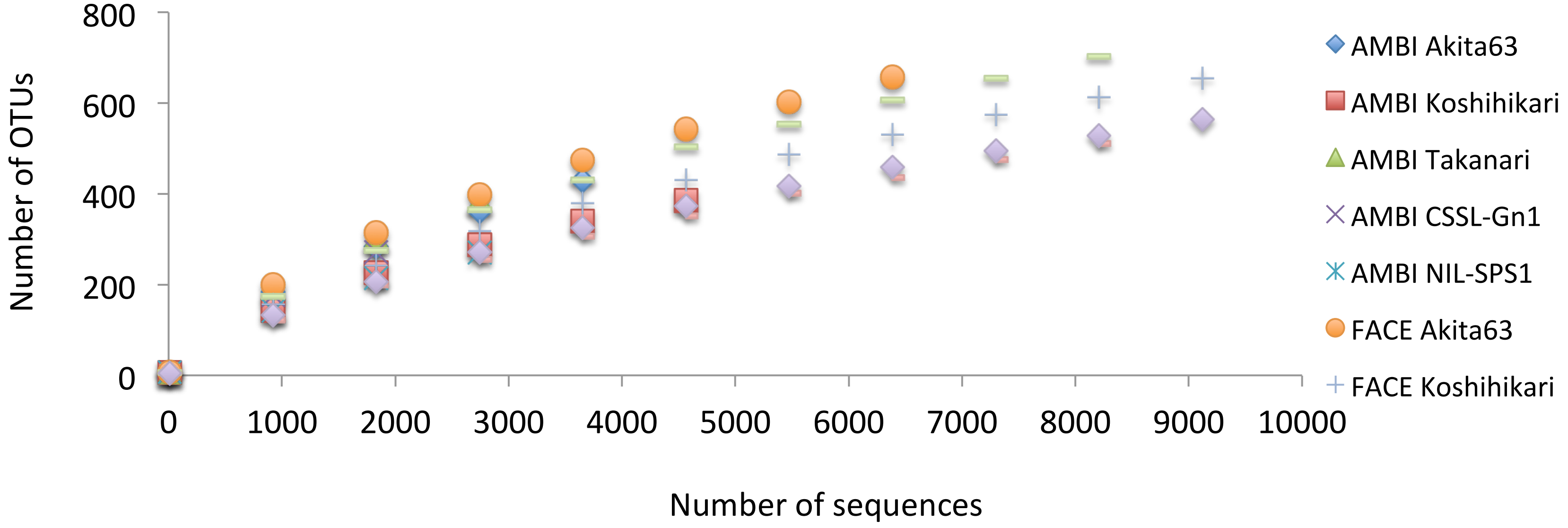

Supplementary Figure 1. Rarefaction curves for each rice genotype and [CO_2] treatment. Each point represents the average of four replicates.

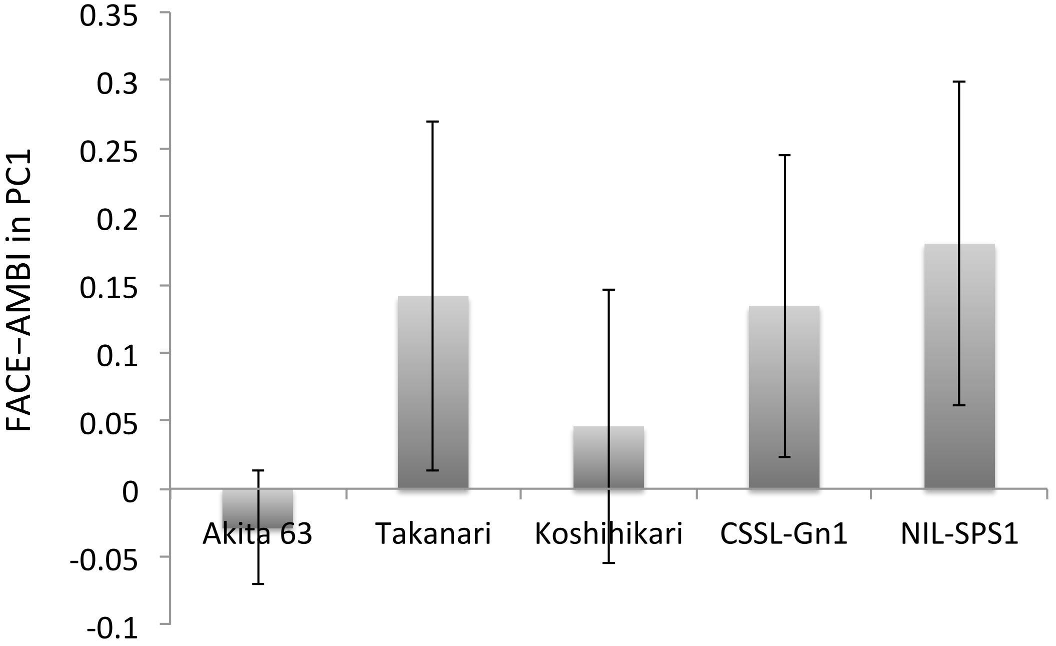

Supplementary Figure 2. The magnitudes of community shift caused by the [CO_2] elevation in each rice genotype. The magnitudes of community shift were calculated from the principal component (PC) 1 of weighted UniFrac principal coordinates analysis plots by subtracting the value of FACE from the value of AMBI in each field (n = 4). Error bars represent standard deviation. Kruskal-Wallis test yielded a *p* value of 0.071. AMBI: ambient levels of CO_2, CSSL: chromosome segment substitution line, FACE: free-air CO_2 enrichment, NIL: near-isogenic line

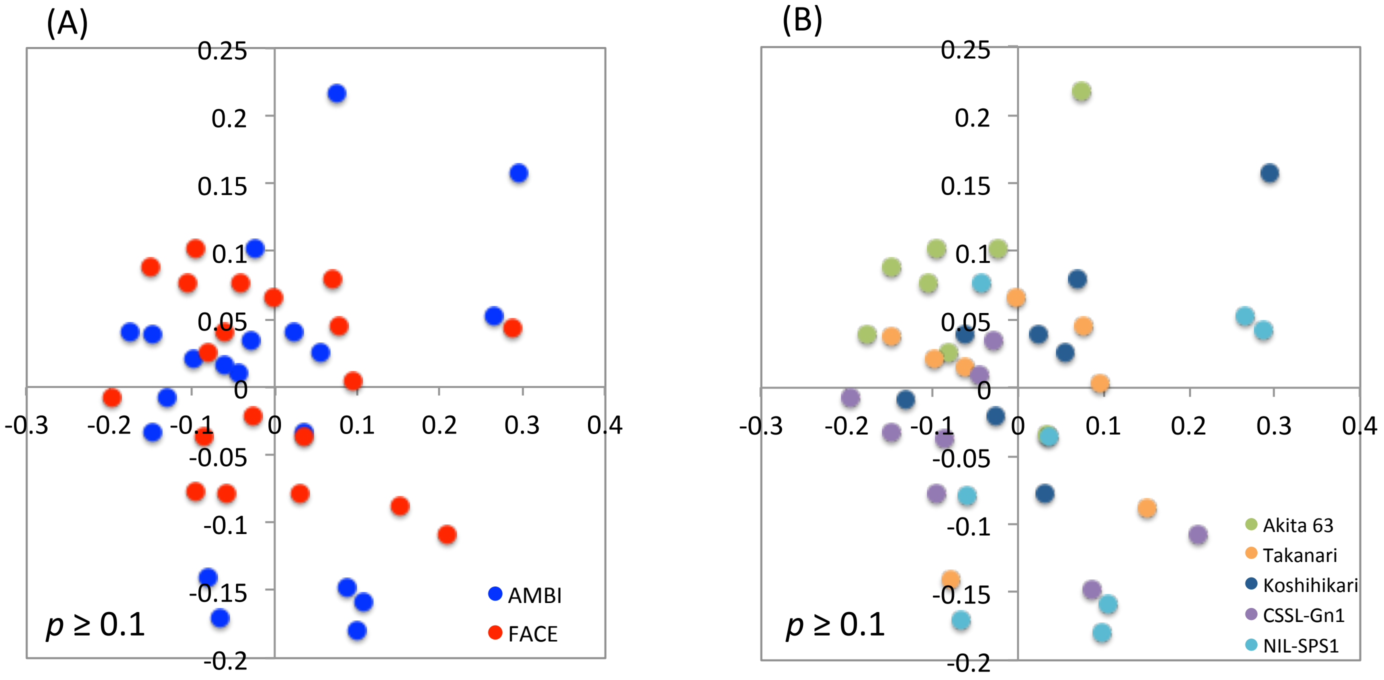

Supplementary Figure 3. UniFrac principal coordinate analysis plots illustrating the effects of the [CO_2] elevation and rice genotype on the structure of root-associated bacterial communities. Distance matrices were defined by an unweighted UniFrac distance. Data points are colored according to [CO_2] treatment in A or rice genotype in B. The results of statistical tests of differences between treatments are indicated in each plot. The [CO_2] × rice genotype interaction did not cause statistically significant changes ($p \geq 0.1$). PC: principal component, AMBI: ambient levels of CO_2, CSSL: chromosome segment substitution line, FACE: free-air CO_2 enrichment, NIL: near-isogenic line

1.2. Supplementary Tables

Table S1 Number of 16S rRNA gene sequences after trimming

	Akita63		Takanari		Koshihikari		CSSL-Gn1		NIL-SPS1	
	Ambient	FACE	Ambient	FACE	Ambient	FACE	Ambient	FACE	Ambient	FACE
Field1	8,783	11,642	9,344	12,080	9,828	12,203	9,225	11,921	8,587	14,395
Field2	9,757	7,273	5,606	8,699	12,665	10,581	9,002	8,681	6,412	10,434
Field3	3,936	6,832	3,121	15,010	5,402	15,598	2,523	9,364	3,112	14,368
Field4	8,681	11,311	8,738	12,246	11,979	13,027	9,129	13,978	9,346	11,486

AMBI: ambient levels of CO_2, CSSL: chromosome segment substitution line, FACE: free-air CO_2 enrichment, NIL: near-isogenic line.

Table S2 Phylogenetic composition (%) and results of statistical tests of rice root-associated bacterial community at the class level

Class	Akita63				Takanari				Koshihikari				CSSL-Gn1				NIL-SPS1				Statistical test		
	AMBI		FACE		AMBI		FACE		AMBI		FACE		AMBI		FACE		AMBI		FACE				
	avg	SD	avg	SD	avg	SD	avg	SD	avg	SD	avg	SD	avg	SD	avg	SD	avg	SD	avg	SD	[CO_2] elevation	Rice genotype	[CO_2] elevation × Rice genotype
Alphaproteobacteria	52.9	9.7	54.3	6.0	53.4	18.0	38.0	7.1	52.6	12.0	47.8	6.7	55.8	13.0	38.8	7.2	70.0	15.6	40.0	9.3	0.085	0.078	**
Betaproteobacteria	22.7	6.2	17.2	2.5	24.1	18.2	48.9	7.5	22.9	7.6	33.9	11.3	20.6	18.5	43.2	6.1	16.0	10.9	41.9	12.0	*	*	*
Gammaproteobacteria	9.6	5.5	11.9	9.8	8.1	3.6	6.0	4.2	13.8	12.1	6.9	6.4	10.2	12.1	4.3	2.0	3.6	1.2	9.8	12.4	ns	ns	ns
Deltaproteobacteria	3.9	3.0	3.5	0.9	3.2	1.0	1.2	0.2	2.5	1.8	2.6	1.3	3.6	1.7	2.9	1.7	1.7	1.2	1.6	1.1	ns	*	ns
Clostridia	1.9	1.0	2.8	0.8	3.4	0.6	0.7	0.2	1.7	1.3	2.6	1.1	2.2	1.4	2.1	1.4	3.4	2.4	1.7	0.6	ns	ns	**
Planctomycetia	2.1	0.9	2.8	0.8	1.9	0.4	1.1	0.2	1.2	0.7	1.5	0.6	1.8	0.7	1.7	0.9	1.0	0.6	1.1	0.6	ns	**	ns
Actinobacteria	1.4	0.2	1.6	0.6	0.9	0.2	0.9	0.5	1.0	0.4	1.1	0.3	1.2	0.5	1.7	0.9	1.4	0.5	0.9	0.3	ns	0.050	ns

Statistically significant effects are indicated: ***p* < 0.01, and **p* < 0.05. Values indicate the probability between 0.05 and 0.1; ns: not significant, AMBI: ambient levels of CO_2, CSSL: chromosome segment substitution line, FACE: free-air CO_2 enrichment, NIL: near-isogenic line, avg; average, SD; standard deviation. Statistical analysis was performed using linear mixed model of the SPSS Statistics software, version 22 (IBM Japan, Tokyo, Japan). [CO_2] and rice genotype were treated as fixed effects, while ring and field × [CO_2] were treated as random effects.

Table S3 Phylogenetic composition (%) and results of statistical tests of rice root-associated bacterial community at the family level

Class	Order	Family	Akita63				Takanari				Koshihikari				CSSL-Gn1				NIL-SPS1				Statistical test		
			AMBI		FACE		AMBI		FACE		AMBI		FACE		AMBI		FACE		AMBI		FACE				
			avg	SD	avg	SD	avg	SD	avg	SD	avg	SD	avg	SD	avg	SD	avg	SD	avg	SD	avg	SD	$[CO_2]$ elevatio	Rice genotype	$[CO_2]$ elevation × Rice genotyp
Betaproteobacteria	Burkholderiales	Burkholderiaceae	20.7	6.4	14.9	2.3	22.3	17.9	46.5	9.4	20.8	6.9	31.6	11.8	18.7	18.2	41.4	5.6	13.7	10.5	40.3	12.3	*	*	*
Alphaproteobacteria	Rhizobiales	Bradyrhizobiaceae	18.6	5.7	18.4	2.4	22.2	6.3	17.8	7.0	16.1	4.0	18.7	4.1	26.0	6.2	21.6	5.3	30.8	11.4	15.5	2.7	ns	ns	*
Alphaproteobacteria	Rhizobiales	Rhizobiaceae	11.9	6.6	13.0	6.3	10.7	9.6	7.1	4.1	18.5	12.7	12.8	9.3	10.6	5.5	5.6	1.1	15.2	1.4	10.6	3.3	ns	0.082	ns
Alphaproteobacteria	Rhizobiales	Methylocystaceae	13.0	3.9	12.8	3.5	9.1	3.8	5.9	2.1	8.7	4.4	8.0	2.8	10.3	4.5	5.4	1.5	12.7	4.0	5.7	2.9	0.057	*	ns
Gammaproteobacteria	Enterobacteriales	Enterobacteriaceae	2.1	0.8	7.6	10.5	3.9	3.9	2.5	2.4	4.4	7.7	0.5	0.3	4.3	6.2	0.9	1.2	1.9	1.5	0.7	0.5	ns	ns	ns
Gammaproteobacteria	Xanthomonadales	Xanthomonadaceae	2.1	3.8	1.3	2.4	1.1	1.8	0.6	0.8	7.1	11.5	4.0	6.5	3.2	5.6	0.4	0.4	0.5	0.9	7.1	13.0	ns	ns	ns
Alphaproteobacteria	Rhizobiales	Hyphomicrobiaceae	1.5	0.2	1.6	0.5	2.7	1.0	1.4	0.3	1.0	0.6	1.0	0.2	1.6	0.3	1.3	0.4	2.0	0.3	1.2	0.6	*	**	*
Alphaproteobacteria	Sphingomonadales	Sphingomonadaceae	2.0	1.2	1.3	1.1	1.2	0.6	0.8	0.6	3.7	5.2	2.2	1.1	1.0	0.5	0.4	0.2	0.6	0.4	1.3	1.0	ns	0.064	ns
Gammaproteobacteria	Legionellales	Coxiellaceae	1.4	0.5	1.3	0.4	1.7	0.6	0.9	0.2	1.2	0.9	1.2	0.5	1.5	0.6	1.5	0.6	0.5	0.4	0.9	0.4	ns	*	ns
Planctomycetia	Pirellulales	Pirellulaceae	1.5	0.7	2.0	0.7	1.3	0.2	0.7	0.2	0.8	0.5	1.0	0.4	1.4	0.5	1.3	0.6	0.7	0.4	0.8	0.5	ns	**	ns
Alphaproteobacteria	Rhizobiales	Phyllobacteriaceae	1.4	0.4	1.3	0.6	1.5	0.9	1.1	0.5	0.5	0.3	0.4	0.1	1.0	0.5	0.8	0.2	1.5	0.8	0.8	0.5	ns	***	ns
Alphaproteobacteria	Rhodospirillales	Rhodospirillaceae	0.9	0.2	1.1	0.3	1.2	0.4	0.8	0.4	0.7	0.4	0.8	0.4	1.3	0.2	1.1	0.3	1.0	0.3	1.2	1.3	ns	ns	ns

Statistically significant effects are indicated: ***$p < 0.001$, **$p < 0.01$, and *$p < 0.05$. Values indicate the probability between 0.05 and 0.1; ns: not significant, AMBI: ambient levels of CO_2, CSSL: chromosome segment substitution line, FACE: free-air CO_2 enrichment, NIL: near-isogenic line, avg; average, SD; standard deviation. Statistical analysis was performed using linear mixed model of the SPSS Statistics software, version 22 (IBM Japan, Tokyo, Japan). $[CO_2]$ and rice genotype were treated as fixed effects, while ring and field × $[CO_2]$ were treated as random effects.
